# Retrospective observation of 6-month survival following decompressive craniectomy in a London major trauma and stroke centre

**DOI:** 10.1186/cc10916

**Published:** 2012-03-20

**Authors:** J Dawson, P Hopkins, J Ling, D Walsh, C Tolias

**Affiliations:** 1 King's Health Partners, London, UK

## Introduction

This study describes 5.5 years of retrospective data examining hospital and 6-month outcome of patients following decompressive craniectomy (DC). The effectiveness of DC remains uncertain with conflicting results in patients with TBI and stroke [1,2].

## Methods

Data were drawn (1 January 2006 to 30 June 2011) from three hospital databases following approval by the institutional board.

## Results

There were 2,148 neurosurgical admissions with 71 undergoing DC. Forty-eight of 71 (67.6%) survived to hospital discharge and 21/33 in both TBI and stroke groups survived to 6 months. See Table 1.

**Table 1:** 

Year	Neurosurgical/total	Total DC	Hospital survival	6-month survival	TBI	MCA stroke	Other
2006	292/1,839	2	2	2	1	0	1
2007	298/1,652	2	2	2	0	2	0
2008	286/1,563	11	10	8 (1N/A)	7	4	0
2009	493/1,840	18	11	10	7	8	3
2010	505/1,835	21	16	15 (1N/A)	11	9	1
2011	274/918	17	7	5 (2N/A)	7	10	0
5.5-year data	2,148/9,647	71	48	42 (4N/A)	33	33	5

## Conclusion

Survival following DC in this institution compares favourably with published data. Reduced survival in 2011 may be a case-mix effect related to increased tertiary referrals. We will now prospectively collect these data including quality-of-life measures.

## References

[B1] Cooper DJ N Engl J Med 2011 364 1493 1502 10.1056/NEJMoa1102077 21434843

[B2] Hofmeijer J Lancet Neurol 2009 8 326 333 10.1016/S1474-4422(09)70047-X 19269254

